# Nursing leaders' perspectives on research in specialist health services: A descriptive study

**DOI:** 10.1002/nop2.1495

**Published:** 2022-11-30

**Authors:** Kari Toverud Jensen, Peter Forde Hougaard, Unni Knutstad

**Affiliations:** ^1^ Oslo Metropolitan University Oslo Norway

**Keywords:** nursing leaders, qualitative research, research culture, specialist health services

## Abstract

**Aim:**

This study is to gain insight into how nursing leaders perceive their contribution to research‐based knowledge in hospital settings.

**Design:**

The study has a qualitative descriptive design.

**Methods:**

Nine nursing leaders were interviewed. Data were analysed based on Braun and Clarke's thematic analysis.

**Results:**

Three themes were developed: the primacy of management and practicalities, delegated responsibility and lack of research competence. Even though the nurse leaders wish to be professional leaders, they seem to prioritize the day‐to‐day management. The nurse facilitators have been delegated, by the nurse leaders, the responsibility for the departments and the employees’ professional development. The participants reported that neither their own leaders, the nurses, nor they themselves had the necessary knowledge or the interest in engaging in research.

**Conclusion:**

There seems to be a lack of awareness, knowledge and priority of nursing research in nursing leadership and absence of a culture of research.

## 1 BACKGROUND

University hospitals are responsible for patient treatment and care, education of health personnel, research and training of patients and next of kin (The Specialist Health Services Act, 1999). Nurses account for a large part of the total number of employees in somatic specialist health services. In the quest for high‐quality health care, nurses represent one group of important actors.

Requirements are set for the patient to receive the best treatment and care based on research, knowledge and current guidelines. For the health service to be research‐based, it is of crucial importance that the nurses participate in the implementation of research. Knowledge produced in a research context cannot necessarily be transferred to clinical activities (Gilje, 2017). Over the past 20 years, evidence‐based practice has received increasing attention as “a modern imperative that is synonymous with qualitative health care” (Bianchi et al., 2018, p. 929). Heggen and og Engebretsen (2009) point out that evidence‐based practice (EBP) has a dominant benefit‐oriented view of knowledge, while in nursing research a broader concept of knowledge has been established. However, it is in the interests of both the profession and the patients that the treatment and practice of nursing are based on research, so that chance and differential treatment are avoided, and the chosen measures are tested and confirmed to have treatment effect. Based on the development of the nursing profession and the increased publications of nursing research, there is a curiosity concerning how new knowledge is implemented in clinical practice.

There is also an increased attention being paid to which processes are necessary to implement research in practice (Gifford et al., 2018; Nilsen & Bernhardsson, 2019). Nilsen and Bernhardsson (2019) show how contextual factors such as access to the organization's economic resources, social relations and leadership play a role in the implementation of research. Gifford et al. (2018) point out the importance of the leader being close to the person who is going to practise nursing, while Sandström et al. (2011) also emphasize that the leader has an important role in influencing the organizational culture in the implementation of research. They claim that leadership is crucial and that the characteristics of the leader, the organization and the culture, are fundamental in building an environment open to implementation. Several researchers emphasize the workplace culture as an important part of implementation of EBP and research (Berthelsen & Hølge‐Hazelton, 2016; Bianchi et al., 2018; Kueny et al., 2015).

Other, such as Kueny et al. (2015), interviewed nurse managers in hospitals about EBP. They described three factors that were considered barriers or facilitators to empowering nurses to use EBP: workplace culture, structure and resources. They pointed out that “Workplace culture, structure, unit‐level resources, availability of institutional resources, and institutional prioritization and expectation of EBP implementation supported” (p. 38) were important for nurse leaders in implementation. A meta‐synthesis by Clavijo‐Chamorro et al. (2022) of nursing managers’ leadership‐related experiences of implementation, showed three factors that facilitate the process. The first factor was teamwork, based on effective communication between nurse managers and staff nurses. The second factor was organizational structures, based on leaders combining management and clinical experience, had awareness of the impact of improved outcomes, and did quality assessment of implementation interventions. The third factor they emphasized was transformational leadership shown by influence on evidence application and, readiness for change among leaders.

Reichenpfader et al. (2015) carried out a systematic review of management in evidence‐based practice in health professions. They found that good leadership enabled the implementation of research and that leaders’ support was an important factor for the implementation's success. Birken et al. (2018) did a systematic review of middle mangers’ role in implementing evidence‐based practice and found that they had an important role in shaping implementation climate. Lunden et al. (2021) studied nurses’ perceptions of their readiness for evidence‐based practice. They revealed that the weakest aspects of leadership were finding resources, promoting discussion, ensuring competency and being visible in knowledge management.

Research has, as pointed out, emphasized the leaders’ responsibility in contributing to evidence‐based practice. However, Harvey et al. (2019) found that nursing leaders lack specific training or research education and possess a more generalist knowledge related to leadership and change management. Still, there seems to be a lack of research concerning nurse leaders’ perspectives on these issues.

## 2 AIM

The aim of the study is to gain insight into how nursing leaders perceive their contribution to research‐based knowledge in hospital settings.

## 3 METHOD

This study is a descriptive qualitative study based on individual interviews of nursing leaders in the specialist health service. A qualitative research design is particularly suitable for gaining insight into and understanding of the informants’ experiences and thoughts on management and research and is suitable for delving into a topic that is little explored (Polit & Beck, 2021).

The interview guide (see Table 1) was developed by the first and last authors, based on previous research and related to topics such as leader philosophy; leaders’ knowledge of their employees and their competence; the research purposes leaders viewed as relevant, relevant standardized guidelines procedures; implementation of new knowledge and the role of leaders. The interviews were carried out at the participants’ workplace except for one participant who was interviewed by phone. Each interview lasted on average 42 minutes (38–52). The questions were asked in such a way as to encourage the participants to share their own experiences. The interviews were recorded digitally and transcribed verbatim by first and last author, but de‐identified.

**TABLE 1:** Examples of themes in the interview guide

Responsibility as a leader in specialist healthcare service The most important part of being a leader In what way is the term evidence‐based practice a suitable expression for your ward's practice Experiences with implementation of research in clinical nursing The relevance of new knowledge and research in clinical nursing How do you implement research or standardized guidelines in your ward and what is your role

### 3.1 Recruitment

We contacted two university hospitals in Norway to recruit nursing leaders. The selection of leaders took place through a “gatekeeper” at the university who had contacts at various hospitals. The request was sent to leaders in the wards who in turn recommended informants, whom the researchers contacted directly, using snowball‐recruiting. Nine female leaders in the specialist health service wanted to participate in this study and were interviewed in December 2020. All informants had nursing backgrounds and represented different wards in the two hospitals. They led either single wards (direct leadership) or larger units with several wards (indirect leadership). The study's participants varied in leadership experience from 2 to 21 years, most of them had more than 5 years’ experience. All of them had either post‐qualifying education or internal management training. Four participants had a master's degree in leadership, management, or health administration and one participant was pursuing a master's degree (see Table 2).

**TABLE 2:** Participants

Participants	01	02	03	04	05	06	07	08	09
Direct/indirect leadership (D/I)	I	D	I	I	D	D	D	D	D
Employees – responsibility	btw 50–75	btw 30–49	btw 30–49	btw 20–29	btw 20–29	btw 50–75	btw 50–75	btw 30–49	btw 50–75
Type of ward	neonatal intensive care	surgery care	medical care	medical care	medical care	surgery care	surgery care	surgery care	surgery care
Internal management training	X	X	X	–	–	X	X	X	–
pqe	–	–	pqe	pqe	pqe	–	–	pqe	pqe
MA	–	MA	–	MA	–	MAwip	MA	–	MA

Abbreviations: MA, master's degree in leadership/management/health administration; MAwip, Master, work in process; pqe, post‐qualifying education.

### 3.2 Analysis

The data were analysed using thematic analysis based on Braun and Clarke's (2006) six steps. The purpose of thematic analysis is to identify patterns and themes in qualitative data. As the first step we read and re‐read the data repeatedly to become familiar with the data. We generated codes during the reading, structuring data in a meaningful and systematic way. Then we constructed themes and saw whether the themes fit together. At step four we modified, developed and revised the themes. As the last step we refined the specifics of each theme, and the overall story (see Table 3).

**TABLE 3:** Themes and subthemes

The primacy of management and practicalities	Delegated responsibility	Lack of research competence
Wanting to be professional leaders, but day‐to‐day management was given priority	Responsibility for professional updating, certification, updating and revising guidelines and procedures (patient safety)	Leaders’ awareness of potential areas for nursing research
Managing, mentoring and supervising the staff	Responsibility for internal dissemination of knowledge to the nursing staff	Lack of knowledge about research
	Responsibility for training, competence plan	Medical‐led research: nurses as facilitators. Nursing research is not prioritized

### 3.3 Ethical considerations

The study was assessed and evaluated by Norwegian Centre for Research Data (NSD) (project nr. 823512). Nurse leaders received both written and oral information about the study and signed an informed consent. The nurse leaders were informed that they were free to withdraw at any time during the study. The data were anonymized, stored in agreement with current guidelines at the university and in line with General Data Protection Regulation (GDPR).

## 4 RESULTS

Through our analysis of the interviews, we developed three main themes: the primacy of management and practicalities, delegated responsibility, and lack of research competence.

### 4.1 The primacy of management and practicalities

The nursing leaders refer to that the administration tasks are all‐encompassing and takes time and attention away from professional leadership. Many of the nursing leaders expressed a desire to do clinical nursing work in the wards, as one of them expressed.

I wish I could be out in the ward and follow up on what they're actually doing. With all the administrative tasks, I can't do that. So, if I'm lucky, if I get to a round in the ward, I talk to them and hear a little bit of how things are going. I have a 3‐minute morning meeting every day to greet them and get them going.(P9)

Staff management responsibilities demanded a large part of their attention as leaders. One of the participants expressed it this way:

… we operate 365 days a year, full package, there is no….quiet times of the year…I'm probably trapped by the fact that we're a department with a very high turn‐over. So, there is a lot of administration due to that, a lot of calls, new nurses coming in, getting them into the system, getting them started. Then it is starting all over again. So, it affects me….I would probably say as of now, a large part of my job is administration.(P6)

This quote also points towards a desire for something more, as several participants also pointed out. One of the leaders put it this way:

I would love to have someone who did the management things for me, so I could perform professional leadership.(P7)

Even though management took most of their attentions, the nursing leaders emphasized the relational aspect of their leadership: having a good relationship with their employees, but also being part of the working community. They also emphasized the need for the same competence as their employees to gain trust and legitimacy among them. There are at least two dimensions of “being out here”: being an equal part of the nursing community, and second, mentoring and supervising the staff. Thereby getting to know the staff and assess their competencies.

I'm out in the clinic a lot, I can prepare a patient for surgery, I can take out and put in catheters, I can put probes….I find myself quite harmless inside the ward, I can help as I can observe. Obviously, a lot of my time goes to observing…. being very visible out in the nursing station…. then I'll hear what they're talking about and join in to correct, guide, encourage, support and stuff like that.(P2)

Leaders and employees working together might creates less differences, and in that way the leader makes herself “benign” as well as achieve legitimacy among the staff. Motivation, encouragement and support were highlighted by several of the informants as important.

You must motivate them. They need to see for themselves that they reach the goal faster by contributing in their own way. They need greater ownership to their job, to feel they are a part of it all… recognising responsibility for it. I think it is important… that they see the importance of coming to work and making an effort. That they can be a role model for others.(P1)

One informant highlighted individual guidance and conversation as one of the things she emphasized in her leadership.

It's more the individual guidance and I do a lot of it. I think without really realising it. But there are many conversations inside the office, both formal and informal, where it is about giving them guidance to do a good job simply. That they need a little like. “you have to work on this” … “you are very good at this, you have to keep holding on to this”. …. lowering the expectations, a little, because they're so impatient I think, a lot of them. Especially the new ones …(P8)

These findings show that even though the nurse leaders wish to be professional leaders they seem to prioritize the day‐to‐day management of the department. Still, they have an awareness of the importance of the relational leadership, to motivate, support and guide their staff in their daily nursing activity.

### 4.2 Delegated responsibility

Several of the leaders had nurse facilitators employed, with delegated responsibilities for updating the unit's nursing professional guidelines and procedures. Even though nurses did cooperate with nursing leaders, much of the work was done without the leader's involvement. Most of the nurse facilitators had extensive experience beyond their nursing education, only a few had a further education or a master's degree.

we chose to have three nurse facilitators. One has 60% to focus on medical technical equipment. … and then we have one who works with auditing procedures…we have one who does bedside guidance. …. they are … open to questions and direct guidance… they are visible and available.(P1)

Another informant underscored these nurses’ responsibility for professional development and review research and that they did most of the professional work with the nursing staff.

…She, who is a nurse facilitator, has a very good system for what they (the nurses) have to learn within the first two years… all the knowledge we want them to acquire. Our designated nurse who is responsible for procedures……must review research and other types of routines to look at what should apply now.(P6)

Several of the nursing leaders described a close cooperation with the nurse facilitators. One leader had a nurse facilitator with a master's degree and derived great benefits from it.

For the professional part I have a nurse who has taken a master's degree quite recently and who is very good in the field of cancer nursing. So, we cooperate… she is very passionate about the subjects, and I have very good use of her.(P5)

One of the informants relied on the nurse facilitators to take care of the professional development of the department, since they were responsible for educational seminars including content and facilitation.

We have two nurse facilitators, which is essential for taking care of the nursing subjects…. I wouldn't have had a chance alone in taking care of the subject in such a good way… …in terms of updating procedures and new changes that happen in the hospital…all the time. The designated nurses are primarily responsible for this.(P8)

Our findings show that the nurse facilitators have been given responsibilities for both professional up‐dates, procedures and compliance training. Also, the employee's development of competencies and the follow‐up appears to be delegated to the professional nurses.

### 4.3 Lack of research competence

The findings show that participants state both that they acknowledge the need for nursing research, but at the same time it is difficult to find the time and resources for these kinds of activities. Furthermore, they underscored that research should be practical and directly applicable to their clinical work.

We had a wound treatment project. We examine the patients before and after a wound intervention and we see afterwards that we have done lots of other things than just documenting changes. We've been too practical, and that's what we become, right? So we're closing that project and we're going to re‐apply and be much more structured… but we're not there in our everyday activities … or we don't have much time to spare so we can sit and philosophize and land a research project, seek funds and things like that. We're not there, but we should have been there, we are a highly competent department that could clearly do much more research.(P2)

Several of the informants had challenges in specifying what they do to contribute to research projects and make use of current research. When asked about their views on research, the participants often turned their rhetorical attention to their nurses’ “lack of interest” or their own superiors. One informant went quite a long way in claiming that nurses are more concerned with experience than with research.

I believe a lot of people think about how we can make things better for the patient, without connecting it to research or something evidence based. Without seeing it with your own eyes and experience it. It's not what you've read in an article … but “I believe it when I see it myself. Regardless of whether I've read it anywhere”. I sound very categorical, but I feel somewhat discouraged over the lack of engagement among the nurses.(P4)

Lack of interest in research was mutual and nursing leaders also experienced this lack of interest from their own superiors.

…my superior does not care about it (research) at all, he thinks researchers are a bit like a fly in the ointment. Because then one doesn't get to produce as much.(P2)

Several of the informants claimed that nurses are not particularly interested in research and that it must be readily available, and that no time has been set aside for nurses to do research. Furthermore, they claimed that most nurses have a lack of interest in doing research. At the most they want to have research results delivered to them without effort, through Facebook or equivalents.

If we had any aspiring candidates – if they had thoughts about and wanted to do research…. If anyone wanted to, I think we'd have made it. We don't have anyone as of now.(P6)

The lack of knowledge of how to do, and how to use, research, was acknowledged by the leaders, and evident in what they stated when talking about their understanding of research. Some of the informants did not have a master's degree and reflected on if they had, they would have experienced research as much easier to deal with. But without a master's degree they think it is too complicated to sort out.

Consistent findings throughout the interviews were that when nurses were involved in research these projects were for the vast majority medically led. In research projects nurses are the physicians’ assistants

…how to express this, (nurses) help them. I don't know if that's exactly the right word, but we do a lot for them to get their research done. … they (nurses) among other things include the patients in the project and then they do some of the procedures …. they help to collect samples, and follow up and have controls, lots of monitoring and things I don't know much about.(P7)

The fact that non‐medical research was underdeveloped compared to medical research, was also described by some participants.

One of the things that is very premature is research that is not initiated by physicians, but other health sciences.(P3)

Even though the findings presented until now for the most part suggest a lack of knowledge and interest, there were signs of developments in the area of nursing led research

… we have a nursing‐driven project… in relation to delirium, but more on the patients, what condition they are in before, during and after delirium…. we do not look at anything medical about it. But looking at function among other things, strength, there are several parameters that one looks at. It's going to be an article and it's going to be the first, so we're totally novices…We've been working on it for a long time… we've been doing this for about three years I think.(P9)

Our study shows that leaders acknowledge areas for nursing research. However, the participants reported that neither their leaders, the nurses, nor they themselves had the necessary knowledge or, in some cases, were not interested in engaging in research.

## 5 DISCUSSION

Overall, our findings in this qualitative study of nurse leaders’ perceptions of their contribution to research‐based practice in hospital settings, suggest that nursing leaders, to only a small degree, are invested and actively engaged in the research‐aspects of the university hospitals’ legally sanctioned mission statements. This is especially the case for nursing research. Our findings point to three important context‐specific factors that contribute to this: (i) management and practicalities are prioritized, (ii) responsibilities for professional development and research implementations are delegated to nurse facilitators that only to a small degree have the necessary knowledge or time and (iii) there is a lack of research competence, especially in nursing research. In the following, we will discuss why this may be the case.

The nurse leaders interviewed in our study all underlined the importance of having good relations with their employees. The knowledge of the nurse's competence forms a necessary basis for professional development. Sandvik et al. (2019) point out that management is context sensitive and that there is a connection between leadership and workers’ work performance. Furthermore, Martinsen (2016) argues that it is important to invest in good relations and visionary leadership. On the other hand, nurse leaders seem not to include research in their visions. In hospital settings, self‐management is an important factor in being able to carry out independent assessments of patients and treatment (Martinsen, 2016). Self‐management seems to us to be important also for taking the initiative to implement research findings and doing nurse‐led research. The consequence of the leaders’ individualization of responsibility to all nurses to practice in accordance with evidence‐based practice might be a reduction of the collective involvement and interest in research. Furthermore, the nurses working in the wards usually have a bachelor's degree and thereby lack the necessary research competence, and they are therefore not prepared to initiate research activities on their own.

### 5.1 The primacy of management vs. professional leadership

One interpretation of the primacy of management is that even when they are leaders, nurses cultivate pragmatism, the flexible roles of nursing and continue to legitimize the hierarchical status of medicine over nursing, especially in the areas of research. This would be an extension of what previously has been described by Allen and Lyne (1997) as that the nursing profession is infused by a culture of flexibility. This flexibility has been framed as expressions of both the nursing profession's loyalty to the patients and deference to the medical profession. If this is the case, it should not come as a surprise that nursing research, which does not have any immediate impact on the patients’ situation, is given low priority. The long‐term gains of research results are suppressed by the short‐term success of patient management. Still some nurses were engaged in medical research as facilitators or assistants to physicians. This may be seen as another sign of deference to the medical professions, and lack of interest in nursing research and professional development.

### 5.2 Culture of research

Our findings show that nurse leaders do support the notion that research is important. Still, the findings also indicate that nurse leaders do not have the necessary knowledge, resources or the necessary focus to actively contribute to an effort to build a research culture. An intellectually stimulating leadership is an especially important driver in developing self‐management and a creative climate among those who are led. The establishment of research cultures depends on both independent initiatives among the staff, sufficient resources (i.e. time and organizational leeway) and focused leadership. According to Kueny et al. (2015), culture, structure and resources are critical factors. Consequently, the stance and orientation of the leaders may be essential in implementing changes impacting rank‐and‐file staff. Our understanding is that successfully building a research culture will depend on the nursing leaders’ active contributions, which we barely observed in our study.

Berthelsen and Koreska (2021) state that in order to develop a practice‐related culture for research in hospital settings, one should focus on research from, by and for practice. Furthermore, they point to the importance of having clinical academic nurses in these settings with master's or Ph.D.‐degrees, to be able to build this culture of research. Our study indicates that there is a lack of academic nurses in the ward settings, and that this makes the leaders’ task of creating a research‐culture even more difficult. Kjølsrud (2016) points out changes in the management role following the introduction of new health trusts and New Public Management. Goals and performance management have been introduced, and finances and control have become important parts of the leadership role. The administrative system might in a greater extent control the leader's everyday life, and this may cause the cultural affiliation with nurses as a professional group to weaken.

However, this does not imply that the situation is without hope of improvement or change, as our study also revealed a growing interest in research and development projects in nursing science, and that the leaders have many ideas of subjects to investigate. We know from other countries that building a culture for nursing research takes decades. In Norway, we have had an active nursing research community since 1980s. However, the clinical focus was strengthened in the beginning of 21st century (2019). Canada has a long history of evidence‐based practice. They have successfully developed an appropriate infrastructure for strategic and clinical levels, and facilitative and managerial leadership (Harvey et al., 2019). Considering these experiences and our findings, we conclude that implementing nursing research and EBP requires institutional and structural commitment, and this cannot be individualized.

### 5.3 Methodological considerations

The sample of participants is small, which is common in qualitative studies. Qualitative studies do not aim to generalize, but to highlight the participants’ experiences and perspectives. We have therefore highlighted quotes and emphasize variation through quoting all the participants. A limitation might be that we have interviewed only university hospitals leaders, and that all of them were women. Interviews with male leaders may have contributed with other experiences and perspectives. To strengthen the study, we might have recruited more leaders from other universities’ hospitals. Despite the low number of participants, the data is rich, nuanced and seems to reflect a wide range of insights (Polit & Beck, 2021).

## 6 CONCLUSION

This study revealed that the nursing leaders interviewed had minimal engagement in nursing research. The reasons we found were a lack of awareness and priority, a lack of knowledge, and the absence of a culture of research. The participants referred to a hospital leadership that did not request or demand nursing research as a base for nurses’ clinical practice. But we also revealed an expectation from nursing leaders to nurses about an individual commitment to research, at the limit of nursing leaders’ responsibility. To the extent that nurses participated in research, it was as facilitators for medical researchers, and on their terms. While previous research has pointed to factors necessary to the successful implementing of nursing knowledge and research in clinical practice, such as leader involvement, research culture and organizational support, we have found that these factors for the most part seems to be missing. A recommendation for further research might be to systematically search for settings where nursing research culture and organizational support actually are present and then analyse the conditions that led nursing leaders to become positive contributors to research‐based nursing knowledge in hospitals settings.

## 7 IMPLICATIONS FOR NURSING MANAGEMENT

The leaders are essential in implementing changes impacting rank‐and‐file staff. Successfully building a research culture depends on the nursing leaders’ active contributions. Implementing nursing research and EBP require institutional and structural commitment and cannot be individualized.

## CONFLICT OF INTEREST

None.
